# Navigating Awareness and Strategies to Support Dementia Advocacy on Social Media During World Alzheimer’s Month: Infodemiology Study

**DOI:** 10.2196/63464

**Published:** 2024-12-27

**Authors:** Juanita-Dawne Bacsu, Sarah Anne Fraser, Ali Akbar Jamali, Christine Conanan, Alison L Chasteen, Shirin Vellani, Rory Gowda-Sookochoff, Corinne Berger, Jasmine C Mah, Florriann Fehr, Anila Virani, Zahra Rahemi, Kate Nanson, Allison Cammer, Melissa K Andrew, Karl S Grewal, Katherine S McGilton, Samantha Lautrup, Raymond J Spiteri

**Affiliations:** 1 Population Health and Aging Rural Research Centre School of Nursing Thompson Rivers University Kamloops, BC Canada; 2 Interdisciplinary School of Health Sciences Faculty of Health Sciences University of Ottawa Ottawa, ON Canada; 3 Department of Computer Science University of Saskatchewan Saskatoon, SK Canada; 4 Department of Psychology University of Toronto Toronto, ON Canada; 5 Toronto Rehabilitation Institute Knowledge, Innovation, Talent, Everywhere Research Institute University Health Network Toronto, ON Canada; 6 Division of Geriatrics Department of Medicine Dalhousie University Halifax, NS Canada; 7 School of Nursing Clemson University Greenville, SC United States

**Keywords:** dementia, Alzheimer disease, advocacy, stigma, myths, awareness, social media, political lobbying, lobbying, X, Twitter, tweet, thematic, promotion, campaign, geriatric, aging

## Abstract

**Background:**

Understanding advocacy strategies is essential to improving dementia awareness, reducing stigma, supporting cognitive health promotion, and influencing policy to support people living with dementia. However, there is a dearth of evidence-based research on advocacy strategies used to support dementia awareness.

**Objective:**

This study aimed to use posts from X (formerly known as Twitter) to understand dementia advocacy strategies during World Alzheimer’s Awareness Month in September 2022.

**Methods:**

Posts were scraped from X during World Alzheimer’s Awareness Month from September 1, 2022, to September 30, 2022. After applying filters, 1981 relevant posts were analyzed using thematic analysis, and measures were taken to support trustworthiness and rigor.

**Results:**

Our study revealed a variety of advocacy strategies, including sharing the voices of lived experience, targeting ethnic and cultural groups, myth-busting strategies, and political lobbying. Although a range of strategies were identified, further research is needed to examine advocacy strategies within different countries and political contexts. Furthermore, the impact of specific strategies on stigma reduction, cognitive health promotion, and policy change needs to be scientifically evaluated.

**Conclusions:**

Our study offers valuable insight into strategies to bolster dementia advocacy and awareness campaigns to support people living with dementia. Findings from our research may provide critical insight for policymakers, organizations, and health professionals working to reduce dementia-related stigma and increase the uptake of risk-reduction activities to support the promotion of cognitive health.

## Introduction

Dementia is an escalating global health concern. Over 55 million people worldwide live with dementia, with nearly 10 million new cases each year [1]. Dementia is the seventh leading cause of death and an important cause of disability [2]. Age is the greatest risk factor for dementia, and as the population ages, the number of people with dementia is expected to increase significantly. By 2050, it is estimated that approximately 153 million people will have dementia worldwide [3]. Although dementia is a common global health issue, stigma and myths about dementia are widespread.

Dementia-related stigma is pervasive and has been documented by numerous literature reviews [4-6]. Stigma refers to a characteristic or attribute that is socially discrediting (in this case, the diagnosis of dementia) and may consist of stereotyping, labeling, loss of status, and discrimination [7]. Studies show that dementia-related stigma often leads to social distancing [8], internalized shame and embarrassment [5], devaluing the lives of people with dementia [9], health care discrimination [10-12], and assumptions that people with dementia should be institutionalized [13]. Consequently, dementia-related stigma creates critical barriers to accessing support and receiving a diagnosis [14].

There are significant misconceptions and negative connotations surrounding dementia. For example, a study of 291 news media articles found derogative wording was often used to describe dementia [15]. Recently, an international survey reported that two-thirds of people erroneously believe that dementia is a normal part of aging [16]. The survey further revealed that 1 in 4 people believe there is nothing one can do about dementia [16]. However, research suggests that approximately 40% of all dementia cases may be prevented through modifiable risk factors [17,18]. Specifically, the World Health Organization published guidelines on 12 modifiable risk factors of dementia ranging from psychosocial factors (eg, social engagement and depression) to lifestyle factors (eg, diet and physical activity) [19]. This research sheds light on the need for more dementia advocacy and awareness to support the promotion of cognitive health.

Globally, organizations are striving to reduce stigma through dementia advocacy and awareness campaigns. For example, the World Health Organization has published numerous global reports advocating for the need to reduce stigma and increase dementia awareness [19,20]. In addition, Alzheimer’s Disease International annually hosts the World Alzheimer’s Day and Month awareness campaigns, as well as publishing annual reports. Despite these initiatives, there remains a paucity of evidence-informed research on specific strategies to support dementia advocacy and reduce dementia-related stigma [4]. This is concerning given that in two separate Dementia Priority Setting Partnerships, older adults living with dementia, care partners, and clinicians emphasized that the top research priorities were to assess effective strategies to target and reduce stigma and discrimination towards persons living with dementia [21].

There is a growing body of literature examining dementia-related stigma on social media [22-26]. For example, a study by Oscar et al [22] used machine learning to identify keywords and phrases commonly used on X (formerly known as Twitter) to perpetuate dementia-related stigma. Another study conducted a content analysis of dementia-related tweets and found stigmatization to be a main theme [26]. In a study of Dutch language tweets, Creten et al [23] identified that dementia-related ridicule contributed to the stigma of dementia on X. Although existing studies focus on documenting dementia-related stigma, there is a paucity of research focusing on specific actions to reduce dementia-related stigma. Consequently, Bartmess et al [25] described the need for further research to examine actions to reduce dementia-related stigma on social media.

Recently, we conducted a study on dementia discourse during Alzheimer’s Awareness Month in Canada that identified educational, promotional, and fundraising tweets being used as actions to enhance dementia awareness [27]. However, this project also shed light on the need for research to examine awareness campaigns outside of Canada to identify advocacy strategies being used to reduce dementia-related stigma globally. Accordingly, the goal of this study is to identify dementia advocacy and awareness strategies used on X during World Alzheimer’s Month in September 2022. Findings from our research may provide critical insight for policymakers, community leaders, and health professionals working to reduce dementia-related stigma among diverse population groups.

## Methods

### Data Collection

From the platform X, we purchased a developer account to acquire access to the official application programming interface (API). The API allows developers to access data from X and create content from the platform. We used the Python-based Tweepy library [28] and X’s API to collect tweets posted from September 1, 2022, to September 30, 2022. The dates selected were based on World Alzheimer’s Month, an international campaign organized by Alzheimer’s Disease International that takes place every September.

Our search terms focused on examining social media discourse during World Alzheimer’s Month. More specifically, our full set of search terms consisted of “World Alzheimer’s Month” OR “World Alzheimers Month” OR “WorldAlzMonth” OR “World Alzheimers Awareness Month” OR “World Alzheimer’s Awareness Month” OR “World Alzheimer’s Day” OR “Dementia Awareness” OR “Alzheimer’s Awareness.”

**Figure 1:**
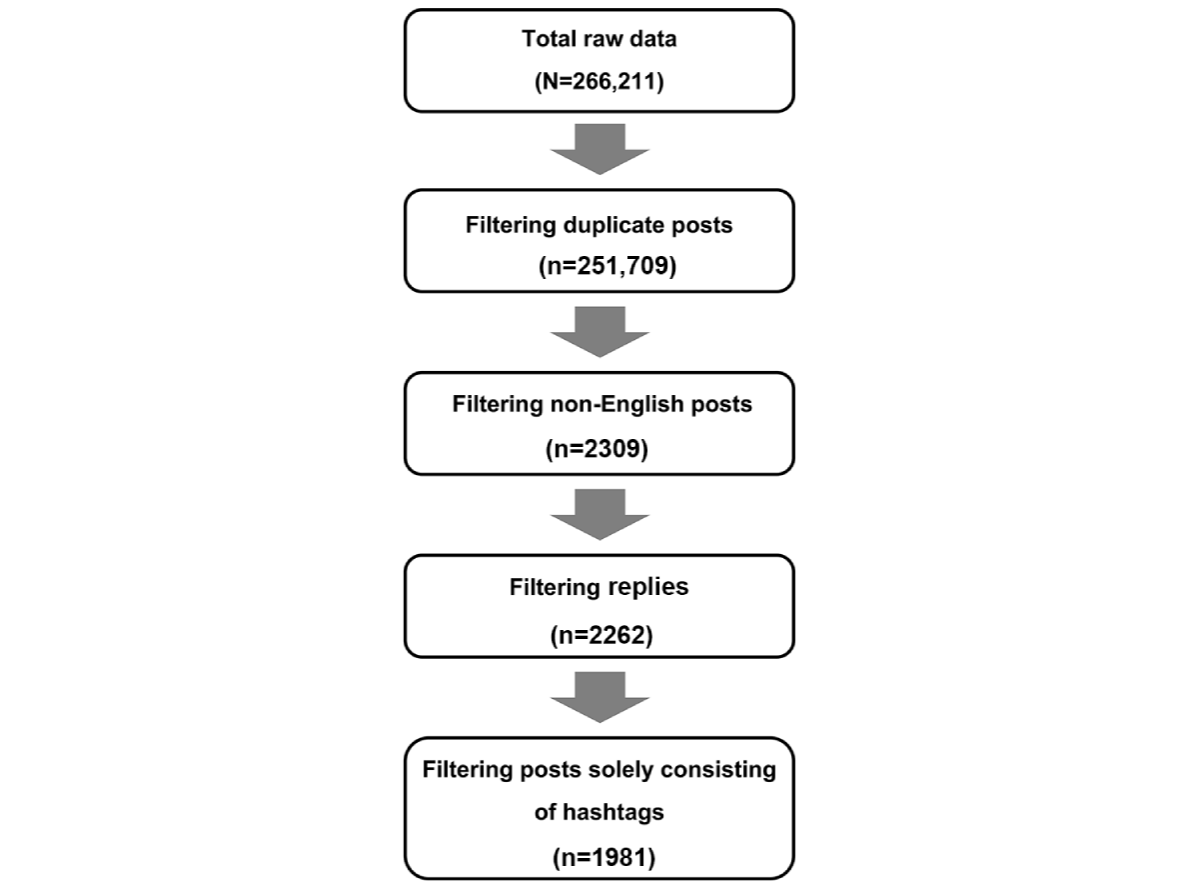
X (formerly known as Twitter) data filtering process.

Our initial search identified 266,211 posts. We removed the posts not written in English (14,502 non-English tweets), which left us with a total of 251,709 remaining tweets. In addition, we removed posts with duplicated content, posts solely consisting of hashtags, and reply posts. We also removed replies because they are often missing important information and contextual content. The data filtering process is documented in Figure 1. The remaining 1981 posts were used for thematic analysis.

### Data Analysis

We analyzed the posts by conducting a 5-stage thematic analysis process outlined by Braun and Clarke [29,30]. This process encompassed (1) reviewing the data, (2) developing initial codes through comprehensive data examination, (3) identifying preliminary themes, (4) refining these themes, and (5) assigning labels to the themes [31]. To analyze the data, we used both inductive (originating from the data, bottom-up approach) and deductive (theory-driven, top-down approach) techniques. First, an initial coding team was formed with 5 of our coauthors who had diverse interdisciplinary expertise and backgrounds, including psychology, computer science, nursing, and population health. This team was formed to develop and pilot test a codebook that was created by reading and reviewing posts (ie, data review and familiarization) to develop an initial list of the codes. To begin the coding process, an inductive bottom-up (data-driven) approach was used, where the researchers created the codes as they analyzed the data. For the pilot test, the coding team independently coded the posts and then compared their codes as a team. A final codebook was created which consisted of 9 codes including (1) stopping stigma, (2) sponsorship (fundraising), (3) simple or basic posts (no educational content), (4) promotional (marketing), (5) lived experiences (family, friend, or person with dementia), (6) fostering stigma, (7) resources (supports), (8) informative (educational messages), and (9) exploitation (capitalistic and political posts). In addition, 2 of our codes (promotional and exploitation) may appear to be similar, so we have provided a description to help differentiate the codes. For example, the promotional code refers to tweets promoting activities, events, conferences, webinars, websites, or informational materials. In contrast, the exploitation code refers to tweets aiming to make a profit from Alzheimer’s Awareness Month for capitalistic gains, such as moving companies, assisted-living homes, or drug companies.

The full team of coauthors participated in practice coding activities using the finalized codebook. The first activity consisted of the trainee practice coding exercise, designed as an introductory task for participants with minimal coding experience. The primary objective was to familiarize trainees with the basic concept of coding and the use of the codebook and its codes. The trainees collectively coded 50 posts. The second practice involved independently coding 75 posts and then comparing individual codes to an answer sheet developed by the initial coding team. The third practice activity involved a team coding exercise in which the full group collectively coded and discussed any coding uncertainties, challenges, questions, or disagreements for 75 posts. After the practice coding was completed, 9 pairs of coauthors (ie, 18 coders) independently coded the same posts (approximately 208 posts per coder) and then met to compare codes and determine the final code for each post through discussion and pair consensus. Any coding discrepancies were resolved through group consensus.

After the coding, the research team used an inductive strategy to sort through the data, aiming to identify prevalent themes by developing a thematic map by grouping the codes into themes. Subsequently, the entire team engaged in a thorough review of the data to ascertain that all relevant themes were captured, clearly articulated, and unique from the other themes [29]. The final stage involved collective discussions within the team to review the clarity and conciseness of each theme name.

### Trustworthiness and Rigor

The criteria created by Lincoln and Guba [32] for trustworthiness were applied in this study to ensure credibility, dependability, and confirmability from data collection to analysis. Credibility was conducted using peer debriefing, member checking, and triangulation. Specifically, credibility was applied through peer debriefing by facilitating practice coding exercises to ensure comprehension of the codes [32]. Furthermore, a codebook was developed to outline the code definitions, keywords, and Twitter examples. This codebook helped to ensure a systematic approach to the coding of the posts. In addition, member checking was implemented to evaluate intercoder reliability by independently evaluating each post by a pair of coders to cross-check the interpretation of the posts [33]. We determined the intercoder reliability by calculating the percentage of agreement between each of the 9 different pairs of coders (ie, 2 coders for each post) and then taking each pair’s percentage of agreement numbers to calculate the overall group average, which was 80% agreement across the coded tweets (Table 1). Less experienced coders were paired with senior coders to demonstrate varying levels of experience with qualitative coding [34]. Researcher triangulation was achieved through the multidisciplinary expertise of the research team [35], composed of experts from various fields such as nursing, nutrition, psychology, public health, gerontology, computer science, and geriatrics. To establish dependability and confirmability, the research process was clearly documented with a comprehensive audit trail [36]. The audit trail was maintained by keeping a record of the research methods and tools, such as the search terms (more details in the Data Collection section), the filtering process, and the thematic map. Regular team meetings with peer debriefing were instrumental in all steps of the research processes, ranging from practice coding activities to theme development.

**Table 1:** Intercoder reliability (weighted percentages).

Coding partners for thematic analysis	Percentage of agreement (%)
KSG and CC	88
RJS and CB	94
SAF and AAJ	52
RG-S and JDB	93
AC and KN	67
FF and KSM	92
MKA and ZR	78
ALC and SV	96
JCM and SL	59
Intercoder reliability average	80

### Ethical Considerations

This study did not require ethics approval because it was based solely on anonymized, secondary data collected from publicly available tweets, which are not classified as human participant research [31,37]. We omitted all usernames and handles before any data analysis to help protect the anonymity of the users. However, we did not anonymize tweet content regarding celebrities, politicians, and national dementia advocates. For example, a new report on ethics and social media research published at the University of Aberdeen notes that celebrities, politicians, and public figures seeking to share their messages as widely as possible do not require anonymity [38]. Accordingly, we did not anonymize posts about celebrity champions (celebrities or athletes), politicians, or nationally known dementia advocates.

## Results

Based on our thematic analysis, 4 main social media advocacy strategies were identified, including (1) sharing the voices of lived experience, (2) targeting ethnic and cultural groups, (3) myth-busting strategies, and (4) political lobbying.

### Strategy 1: Sharing the Voices of Lived Experience

A predominant strategy used for dementia advocacy focused on sharing the voices of lived experience, including people living with dementia and their family care partners. For example, this strategy called on people living with dementia to spread awareness and often shared insights to support dementia advocacy and leadership actions. This strategy is highlighted in the following posts:

Share your story with us this #worldalzheimersmonth

VOICES OF LIVED EXPERIENCE: Author & advocate Myrna Norman shares her insights about how more people living with dementia might be engaged in leadership & research opportunities. Read Advocacy in Action.

World Alzheimer’s Month also enabled people living with dementia to share their lived experiences to improve the general public’s understanding of dementia. These experiences challenged negative stereotypes in describing that it is possible to live well with dementia. Lived experiences often included sharing personal challenges as well as support to enhance the quality of life, ranging from technology to an optimistic mindset.

A #WorldAlzheimersMonth message of support from Patient & Family Advisory Council member:

I am a person living with vascular dementia for 10+ years. I live independently & I live well. Some days can be challenging, but I use technologies to help.

After living with dementia for almost 5 years, I came to realize my life can be good whenever people that I work with practice what I called the Four As - Acknowledge, Accept, Accommodate, and Adapt.

Many celebrity champions (including famous Olympians and actors) used their celebrity status and fame to help build a platform to raise dementia awareness. Examples of celebrity champions included Mariah Bell, Natalie Morales, Selenis Leyva, Izzy Diaz, Oscar Nunez, Seth Rogen, Lauren Miller, Vicky McClure, and Peter Gallagher, who shared their family members' stories and their own experiences as care partners in raising awareness and advocate for people living with dementia.

Actor Peter Gallagher shares his personal experience with Alzheimer disease. As the awareness month nears its end, we should always remember to fight the stigma surrounding Alzheimer’s and raise awareness.

#ENDALZ Celebrity Champion and #TheOffice star Oscar Nunez raises Alzheimer's awareness for his dad and grandmother. Thank you, Oscar, for making a difference in the fight to end Alzheimer's! #HispanicHeritageMonth.

Researchers also shared stories and encouraged people to support research and awareness of people living with dementia.

As #World Alzheimer's Month draws to a close, we are sharing this video of inspirational Research Champion who explains why encouraging others to take part in dementia research is so important to her.

### Strategy 2: Targeting Ethnic and Cultural Group Awareness

Another strategy to enhance dementia advocacy was to specifically target different cultural, ethnic, and linguistic groups. This took the form of raising awareness, highlighting disparities, and using creative hashtags to increase the visibility of the posts. For example, awareness materials and activities in various languages were showcased, reaching and engaging individuals from various ethnic and cultural backgrounds. A wide range of materials also included culturally tailored content, tips, and ideas to raise awareness in diverse communities. The following posts demonstrated ethnically and culturally targeted awareness strategies:

Thank you to all friends and colleagues in Greece for translating the 12 risk factors for #dementia #alzheimers in Greek efkaristo' poli' #worldalzmonth if you are Greek please share!

It’s September It's World Alzheimer's Month These are our list of activities in mix culture – languages [including Dutch, English, and Bahasa Indonesia]… @alzi_indonesia @AlzDisInt

In honor of #WorldAlzheimersMonth we would like to highlight Dr. Tiffany Chow, who shares her insights on the prevalence of Alzheimer's disease among the AANHPI (Asian American, Native Hawaiian & Pacific Islander) community.

Efforts to enhance dementia awareness also included highlighting existing disparities between different cultural and ethnic groups in terms of dementia incidence and access to care.

Older African Americans are twice as likely to have Alzheimer’s or other dementia as older white Americans, while older Hispanic adults are 1.5 times as likely to have Alzheimer's. Awareness and education are vital.

Posts also incorporated the usage of hashtags to target specific cultural and ethnic groups, such as “#HispanicHeritageMonth.” In addition, partnerships with cultural organizations were also used to promote awareness. These hashtags and partnership strategies are highlighted in the following posts:

We are especially grateful for the wonderful partnership of @ProgresoLatino in helping to raise Alzheimer's awareness among the Latino community in our area. Together, we are making a difference. #HispanicHeritageMonth #AlzRI 5/5

Happening today 9/24 at Noon #WorldAlzheimersMonth #HispanicHeritageMonth #ENDAlz

As such, culturally and ethnically targeted awareness materials and tips increased the accessibility of knowledge and available support and may resonate better within different groups.

### Strategy 3: Myth Busting

Another dementia advocacy strategy was to address misinformation about dementia. Most examples included educational-style lessons or posts alluding to evidence or quotes from research related to treatment options or symptoms of Alzheimer disease. This myth-busting related to education is demonstrated in the following posts:

Many people still wrongly believe that dementia is a normal aging. This alone highlights how important public awareness campaigns, like World Alzheimer’s Month, are for changing perceptions and increasing public knowledge around dementia and Alzheimer disease.

This year the theme for World Alzheimer’s Month is “Know Dementia, Know Alzheimer’s”. Dementia is an umbrella term for a collection of symptoms caused by disorders affecting the brain & impacting memory, thinking, behaviour & emotion. The most common is Alzheimer's disease.

Many tweets focused on combating myths and false assumptions about dementia. For example, these tweets would often challenge dementia myths by providing facts, information, and statistics about dementia. The following are related examples:

Myth - dementia does not just affect older adults! Age is a risk factor - but in rare cases, it affects younger adults too... Scientists estimate that 260 in 100,000 people (aged 30-64) develop early onset dementia!

Myth - there are no treatments available for people with Alzheimer’s disease! There has been lots of progress on treatments for people with Alzheimer’s, including medications and coping strategies to help manage behavioral symptoms.

### Strategy 4: Political Lobbying

Political lobbying of governments was also used to increase dementia advocacy. This strategy often included lobbying members of the government to make dementia a national priority. For example, this strategy included targeted political advocacy to support the development of national dementia plans for countries without any national plan. This political lobbying for national dementia plans is highlighted in the following posts:

When countries have a national #dementia plan, they are more likely to have robust strategies for post-diagnostic support in place. During #WorldAlzMonth, #KnowDementia & #KnowAlzheimers better by reading about the importance of national dementia plans.

…EmmanuelMacron France now does not have a dedicated national dementia plan.

Political lobbying also included advocating for increased government support and funding to enhance dementia research. For example, posts highlighted the need for larger clinical trials with diverse populations to support dementia research. This advocacy for government funding to support dementia research is demonstrated in the following posts:

A call for the government to reduce #health #inequalities in #dementia - from prioritizing funding for scientific innovation, through to facilitating larger and more diverse dementia trials

Will you lend your voice for research? This #WorldAlzMonth, we're asking MPs across the UK to write to @trussliz [the Prime Minister of the United Kingdom in 2022], calling for people living with dementia & research… to remain a political priority. Write to your MP now…

## Discussion

### Principal Findings

Our study examined advocacy strategies being used during World Alzheimer’s Awareness Month. This study is among the first to examine dementia advocacy used in a global awareness campaign on social media. Understanding advocacy strategies is essential to improving dementia awareness, reducing stigma, supporting cognitive health promotion, and improving policymaking for people living with this condition and their care partners. Our study revealed a variety of advocacy strategies, including sharing the voices of lived experience, targeting diverse ethnic and cultural groups, myth-busting strategies, and political lobbying.

Our study identified that political lobbying was a key strategy used to conduct dementia advocacy with governments. However, it is important to note that the political context differs by country and often requires different advocacy efforts. For example, our study found that some countries without national dementia strategies (eg, France) tended to focus on the issue of advocacy by endorsing their governments to develop a national dementia plan, whereas other lobbying initiatives focused on financial advocacy and lobbying governments for increased funding and resources to support dementia research. Consequently, further research is needed to examine the political climate and policy-related issues that are relevant to supporting dementia advocacy within different countries, jurisdictions, and political contexts.

Other strategies identified in our study involved myth-busting, targeting different ethnic and cultural groups, and sharing lived experiences. These strategies focused on increasing dementia awareness and education among the public, including diverse groups. Seetharaman and Chaudhury [39] note that sharing lived experiences of dementia helps to challenge common stereotypes and misconceptions about dementia. In particular, this study identified celebrities (including famous athletes, Olympians, and actors) and national dementia advocates sharing their voices of lived experience to challenge the stigma of dementia. This finding contrasts with our previous research [27] that reported a lack of lived experience and celebrity advocates during Alzheimer’s Awareness Month in Canada. Through celebrity endorsement and the voices of lived experience, people living with dementia and care partners can formulate a collective identity to bring about social change and networking opportunities to challenge dementia-related stigma [40]. Consequently, Weetch et al [41] note that dementia advocacy may offer well-being benefits for those who engage through the activity itself and the development of new social networks.

Similar to existing research in mental health [31] and dementia [27], our study found several cursory posts that made basic reference to the awareness campaign. Although these posts supported basic awareness, they also shed light on the opportunity for more targeted advocacy strategies to enhance dementia education, counter misconceptions, and reduce dementia-related stigma [27]. Consequently, having more targeted posts may decrease the risk of having posts being disregarded and perceived as pointless.

### Limitations

Although our study was conducted in a comprehensive manner, it is not without limitations. More specifically, our study focused solely on textual data and excluded images and photos on X. However, this exclusion limits our study’s ability to draw conclusions based on image content to inform dementia advocacy and awareness-raising efforts. For example, a recent study suggests that a post with an image contributes to higher user engagement on social media [42]. This research suggests that posts with high-quality, professional images and human faces are more likely to lead to higher user engagement on the X platform [42]. Further research is needed to examine the impact of images on user engagement during World Alzheimer’s Awareness Month. This research on image content may provide critical insight to inform novel interventions to raise awareness.

In addition, our study did not include any evaluation to assess the impact of the different advocacy strategies. Although a range of strategies was identified, further research is needed to evaluate the impact of the specific strategies on supporting dementia advocacy. For example, research is needed to examine whether these social media strategies effectively work to reduce dementia-related stigma. Moreover, studies are required to assess whether dementia advocacy on social media advocacy improves dementia awareness and advances cognitive health promotion. In addition, further research is needed to examine whether dementia advocacy on social media contributes to and/or results in policy and program change to support people living with dementia.

Another limitation is that our data were restricted to posts in the English language. This limitation is particularly relevant given that approximately 60% of people with dementia live in low- and middle-income countries [43], where English may not be the predominant language. Consequently, this language restriction may have overlooked novel strategies employed to target different cultural, linguistic, and ethnic groups. Accordingly, further research is needed to examine dementia advocacy strategies used in non-English-speaking countries.

### Conclusions

Our study examined dementia advocacy strategies used during World Alzheimer’s Awareness Month in September 2022. Understanding advocacy strategies is vital to reducing dementia-related stigma, countering myths, and supporting the promotion of cognitive health. Furthermore, advocacy is important to ensuring that dementia care is prioritized and supported by governments. By examining different advocacy strategies, our study offers valuable insight into ways to bolster dementia advocacy and awareness campaigns to support people living with dementia.

Our study identified a variety of advocacy strategies ranging from myth-busting to political lobbying. Although a range of strategies was identified, further research is needed to examine advocacy strategies within different countries and political contexts. Furthermore, evaluation research is needed to assess the impact of specific strategies on stigma reduction, cognitive health promotion, and policy change. Findings from our study may provide critical insight for policymakers, researchers, and health professionals working to reduce dementia-related stigma and increase the uptake of risk-reduction activities to support cognitive health promotion.

## Abbreviations

API: application programming interface

## Data Availability

The data sets generated during and/or analyzed during this study are not publicly available but are available from the corresponding author on reasonable request.
